# Demographics, clinical characteristics and neonatal outcomes in a rural Ugandan NICU

**DOI:** 10.1186/1471-2393-14-327

**Published:** 2014-09-19

**Authors:** Anna Hedstrom, Tove Ryman, Christine Otai, James Nyonyintono, Ryan M McAdams, Deborah Lester, Maneesh Batra

**Affiliations:** >Department of Pediatrics, Division of Neonatology, University of Washington, Seattle, USA; Department of Epidemiology, University of Washington, Seattle, USA; Kiwoko Hospital, Luweero, Uganda; ISIS Foundation, Edmonds, USA

**Keywords:** Millennium development goals, Neonatal mortality, Sick newborn care, Infant mortality, Low-resource settings, Global health, Birthweight, Gestational age, Low and Middle Income Countries

## Abstract

**Background:**

Ninety-six percent of the world’s 3 million neonatal deaths occur in developing countries where the majority of births occur outside of a facility. Community-based approaches to the identification and management of neonatal illness have reduced neonatal mortality over the last decade. To further expand life-saving services, improvements in access to quality facility-based neonatal care are required. Evaluation of rural neonatal intensive care unit referral centers provides opportunities to further understand determinants of neonatal mortality in developing countries. Our objective was to describe demographics, clinical characteristics and outcomes from a rural neonatal intensive care unit (NICU) in central Uganda from 2005–2008.

**Methods:**

The NICU at Kiwoko hospital serves as a referral center for three rural districts of central Uganda. For this cross sectional study we utilized a NICU clinical database that included admission information, demographics, and variables related to hospital course and discharge. Descriptive statistics are reported for all neonates (<28 days old) admitted to the NICU between December 2005 and September 2008, disaggregated by place of birth. Percentages reported are among neonates for which data on that indicator were available.

**Results:**

There were 809 neonates admitted during the study period, 68% (490/717) of whom were inborn. The most common admission diagnoses were infection (30%, 208/699), prematurity (30%, 206/699), respiratory distress (28%, 198/699) and asphyxia (22%, 154/699). Survival to discharge was 78% (578/745). Mortality was inversely proportional to birthweight and gestational age (P-value test for trend <0.01). This was true for both inborn and outborn infants (p < 0.01). Outborn infants were more likely to be preterm (44%, (86/192) vs. 33%, (130/400), P-value <0.01) and to be low birthweight (58%, (101/173) vs. 40%, (190/479), P-value <0.01) than inborn infants. Outborn neonates had almost twice the mortality (33%, 68/208) as inborn neonates (17%, 77/456) (P-value <0.01).

**Conclusions:**

Understanding determinants of neonatal survival in facilities is important for targeting improvements in facility based neonatal care and increasing survival in low and middle income countries.

## Background

Childhood mortality remains a significant global challenge. Almost 7 million children under 5 years of age die each year, including 3 million newborns in their first month of life [1]. The proportion of childhood deaths that occur in the neonatal period has actually increased from 36% to 43% since 1990 despite improvements in under-5 mortality [1]. This comparatively slow decline in neonatal mortality is a significant barrier to achieving Millennium Development Goal 4, which targets a two-thirds reduction in childhood mortality from 1990 to 2015 [2].

Overall, it is estimated that up to 50% of all neonatal deaths occur within the first 24 hours after birth, and 75% by one week of age [3]. The most common causes of neonatal deaths are infection, prematurity and intrapartum related causes (“birth asphyxia”); the frequencies of these deaths vary between and within regions [4]. Achieving reductions in neonatal mortality globally has been challenging for a variety of reasons, including limited political prioritization of newborn health, inadequate financial commitment to neonatal care by funding sources, and slow scale up of high impact maternal-child interventions.

There is a paucity of information regarding determinants of mortality for newborns in settings with the highest burden of neonatal deaths. Ninety eight percent of neonatal deaths occur in low and middle income countries (LMIC) where most births and deaths happen at home [1, 5]. In these settings, vital registration is either limited or non-existent and information regarding gestational age, birthweight and treatment course are often unknown [6, 7]. Global models of key neonatal health determinants (e.g., birthweight, gestational age, birth location) are derived from countries where vital registration is adequate and extrapolated to regions with poor vital registration [8]. Accurate data regarding causes of neonatal mortality for the majority of the world’s newborns are therefore lacking.

Recently, global efforts to reduce neonatal mortality have focused primarily on community-based interventions largely because the majority of births and deaths occur in the home, and access to facilities with capacity to manage newborns has been limited [9]. These interventions have included training birth attendants to provide neonatal resuscitation and immediate care after delivery and encouraging perinatal hand washing [10, 11]. Other efforts have utilized community health workers to perform home perinatal visits, recognize illness and refer sick neonates, and manage neonatal infections [12, 13]. Such efforts have led to reductions in neonatal mortality ranging from 15 to 85 percent [9]. To further support important community-based interventions and expand life-saving services, improvements in access to quality facility-based neonatal care are required [14].

Neonatal intensive care units (NICUs) have been developed in LMICs and are being utilized to care for high-risk neonates. The majority of such centers are located in urban settings, where NICUs serve more geographically clustered populations with access to specialized care and services. Outcomes from LMIC NICUs provide opportunities to better understand determinants of neonatal mortality and to target interventions with the potential for greatest improvements in neonatal survival. Unfortunately, there are few published reports on outcomes from NICUs in LMICs, particularly from rural settings. In 2003, a study from a tertiary referral NICU in Tanzania reported a mortality rate of 68% among newborns born prior to 31 weeks gestation and 55% mortality among those with a birthweight less than 1 kilogram [15]. Sen et al. described the development of a NICU in a West Bengal district with poor access to facility care and a very high neonatal mortality rate, which resulted in a reduction in neonatal mortality in the hospital from 31% to 25% between 2003 and 2005 [16]. A nineteen-year review of admissions to a rural Kenyan hospital demonstrated a decreased neonatal inpatient case fatality from 31% in 1990 to 17% in 2008 [17]. While these studies reveal valuable insights into the burden of neonatal mortality in LMICs, a major limitation to developing targeted improvements in facility-based care is the lack of information reported regarding maternal, neonatal and clinical characteristics of the patient populations served. This gap in our understanding of facility-based neonatal care and mortality represents a barrier to improving care for newborns in LMICs.

In this study, we describe demographic and clinical characteristics, along with outcomes obtained from a rural facility-based neonatal intensive care unit in central Uganda from 2005 to 2008.

## Methods

### Setting

Kiwoko hospital serves as a rural referral center for three districts of central Uganda (combined population: 600,000) where 31% of births take place at home and only 33% of births are registered with the government [18]. As of 2006, this region had a neonatal mortality rate consistent with other rural areas in Uganda (33 per 1000 live births) and an under-5 mortality rate of 129 per 1000 live births [19]. Kiwoko hospital has 4–8 doctors working at a time and has bed capacity for up to 300 patients.

The NICU in Kiwoko hospital accepts inborn and outborn newborns with gestational ages greater than 24 weeks and up to a chronologic age of 3–6 months. It opened in 2001 as a 20-bed unit with 300 admissions per year and was staffed by one nurse and two midwives [20]. The most commonly reported admission diagnoses at that time were prematurity, neonatal tetanus, prematurity with respiratory distress, and birth asphyxia. By 2012, the NICU had 35 beds, admitted 600 newborns per year, the majority of which were born at the hospital, and was staffed by 23 nurses and an assigned physician. A prospective NICU database containing 203 demographic and clinical variables for all admissions was established in 2005, allowing for more in-depth analysis of patient characteristics and outcomes.

**Figure 1:**
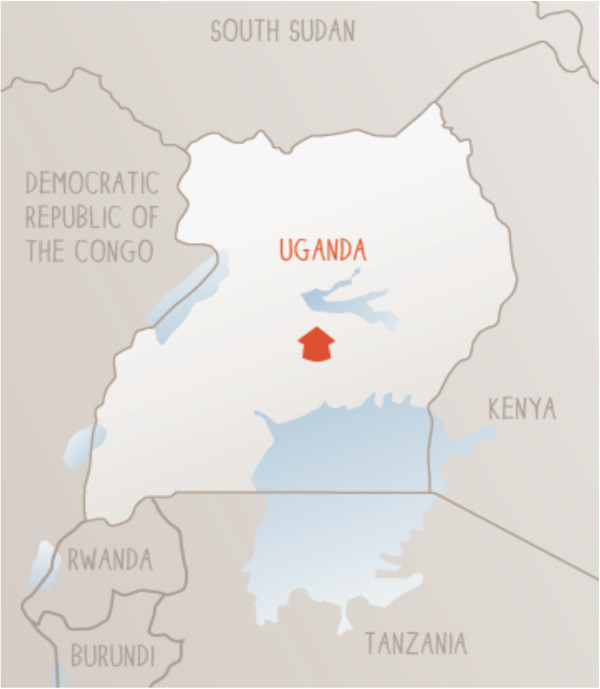
**Location of Kiwoko Hospital, Uganda.** Kiwoko Hospital in Nakaseke district shown with hut symbol (used with permission of the ISIS foundation).

During the period of this study, the NICU had capacity for thermoregulation, intravenous hydration, cup and nasogastric tube feedings, limited phototherapy equipment, limited number of oxygen concentrators for use with nasal cannula, and an intermittent electrical supply. Neonates requiring pediatric subspecialty care required transfer to the national referral center in Kampala, located two hours’ travel by car. (Figure 1) During the study period there was at least one other NICU in Uganda (at the national hospital in the capital city) that had similar capabilities to care for ill neonates.

### Data collection and analysis

This study was conducted by retrospective review of all admissions to the NICU from December 2005 through September 2008. Prior to 2005, no medical information systems existed in the Kiwoko NICU. Beginning in October of 2005 nurses recorded relevant health information at the time of admission for each infant using a medical record form developed for this NICU. The form was then updated throughout the hospitalization by the nursing staff, and completed at discharge/death of the patient. The medical record form included: maternal and delivery characteristics (e.g., maternal age, type of birth attendant), neonatal clinical characteristics (e.g., birth weight, admission diagnosis), hospital course (e.g., blood transfusions, oxygen therapy) and discharge status. Data from all forms during the study period were manually entered in to an electronic database in SPSS.

All analyses reported in this paper are restricted to infants less than 28 days of age at admission. Descriptive statistics were used to assess demographic and clinical characteristics as well as neonatal outcomes. Medians and ranges were calculated for continuous variables. As appropriate, continuous variables were converted to clinically meaningful categories and analyzed as categorical variables. Frequencies were calculated for categorical variables. All analyses were disaggregated by location of birth, with those born at Kiwoko Hospital referred to as “inborn”, and those born outside of the hospital in a surrounding village health post or at home referred to as “outborn”. The chi-square test was used to evaluate differences between inborn and outborn infants and linear regression test for trend was used to evaluate differences in mortality based on birth weight and gestational age group. Gestational age was determined (in order of preference) by the mother’s report of her due date as determined from prenatal care, her last menstrual period or using Ballard assessment at admission if available. When birth weight was not known, admission weight was used if the patient was admitted within 3 days of birth. Admission diagnoses were categorized (such as prematurity, respiratory distress, infection, etc.). A neonate could have multiple admission diagnoses recorded. For admission diagnoses, reported percentages reflect the number of neonates with a particular diagnosis recorded as a proportion of the total number of neonates included in the study. For all analyses the reported unit of analysis is the neonate. The University of Washington Human Subjects Division reviewed the protocol and designated this study Minimal Risk and granted a waiver of consent due to the nature of the retrospective review and inability to contact subjects that were included in the database (HSD Study #43072). Local approval was obtained from Kiwoko hospital. This research has adhered to the STROBE guidelines for cross sectional studies.

## Results

### Subjects

**Figure 2:**
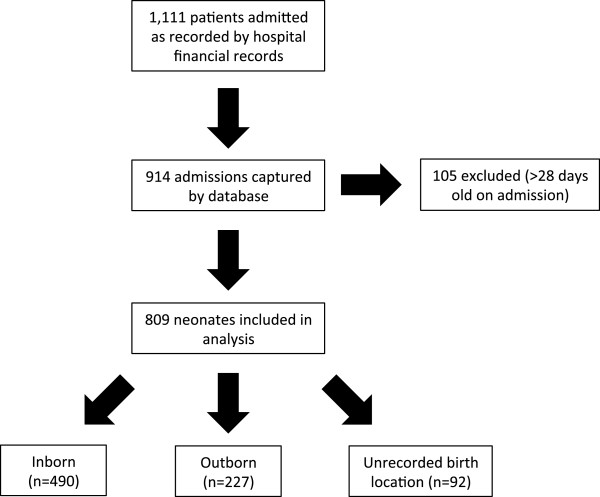
**Patient population.** Number of total patients admitted by hospital financial records, those captured by database and those included in the study.

Between December 2005 and September 2008, 1111 babies were admitted to the NICU as recorded by hospital financial records (Figure 2). Our dataset captured 914 NICU admissions, which represents 82% of those recorded by hospital financial records. 809 of the 914 patients in our database met our inclusion criteria of being less than 28 days of age at admission. Completeness for the individual variables recorded ranged from 27% to 93%. There were 92 neonates (11%) for whom inborn/outborn status was not recorded; therefore these children were not included in the disaggregated analyses. Among children with known birth location, 68% (490/717) were inborn at Kiwoko hospital, and the remainder were outborn.

### Maternal characteristics and infant delivery

Among admitted neonates with available data, the majority of their mothers were between 18 and 34 years old (80%, 405/504) and reported at least one prenatal care visit (96%, 723/755) (Table 1). Overall, 77% of admitted neonates were from singleton pregnancies. Four maternal deaths after childbirth were reported (0.6%, 4/667) in this study population and 2 of these occurred among mothers of inborn neonates. The most common mode of transportation to the hospital was by vehicle (53%, 335/627) and the second most common was by motorcycle (40%, 252/627). Nurses or midwives delivered most infants (58%, 431/747) and doctors delivered 31% (231/747). The majority (68%, 513/753) of deliveries were vaginal. Cesarean section delivery occurred in 46% (214/471) of inborn and 1% (2/212) of outborn births.

**Table 1:** **Maternal and delivery characteristics of neonates admitted to Kiwoko Hospital NICU (December 2005 through September 2008)**

	All neonates		Inborn		Outborn	
	%	(No.)	%	(No.)	%	(No.)
**Mother**						
**Maternal age**						
<18	10.5	(53)	11.2	(33)	9.4	(15)
18-24	40.9	(206)	38.3	(113)	45.0	(72)
25-34	39.5	(199)	41.7	(123)	36.3	(58)
35-39	7.5	(38)	7.1	(21)	8.1	(13)
≥40	1.6	(8)	1.7	(5)	1.3	(2)
Any prenatal care	95.8	(723)	97.4	(444)	95.0	(209)
Maternal death	0.6	(4)	0.5	(2)	0.5	(1)
**Delivery**						
**Mode of transportation**						
Vehicle*	53.4	(335)	53.3	(196)	55.4	(107)
Motorcycle	40.2	(252)	40.0	(147)	39.9	(77)
Bicycle or foot	6.4	(40)	6.8	(25)	4.7	(9)
**Type of birth attendant**						
Doctor	30.9	(231)	45.4	(207)	1.8	(4)
Nurse/midwife	57.7	(431)	54.4	(248)	69.1	(150)
Traditional birth attendant	7.5	(56)	0.0	(0)	20.3	(44)
Family or other	3.9	(29)	0.2	(1)	8.8	(19)
**Mode of delivery**						
Vaginal	68.1	(513)	54.6	(257)	99.1	(210)
Vesarean section	31.9	(240)	45.4	(214)	0.9	(2)
Singleton	77.2	(457)	80.6	(279)	74.2	(141)

*includes: car, taxi, special hire, ambulance.

Each analysis is based on available data for that measure, as such denominators differ depending on the response rate.

### Infant characteristics and hospital course

Males represented 55% (433/785) of neonatal admissions (Table 2) and this did not differ between inborn and outborn admissions. The majority (87%, 633/729) of admitted neonates had a birth weight between 1.5 and 4 kg, while 2% (18/729) were less than 1 kg. Birth weight was estimated based on admission weight for 16% (114/729) of neonates. Overall no birth weight data were available for 10% of neonates (80/809); 24% of outborn neonates (54/227) and 2% of inborn neonates (11/490). Among neonates with available data, the majority (58%, 101/173) of outborn neonates were low birth weight (<2.5 kg) compared to 40% (190/479) of inborn neonates (chi-square = 18.0, P-value <0.01). Gestational age estimates were available for 82% (663/809) of neonates and ranged from 24 to 44 weeks. Forty four percent (86/192) of outborn neonates were preterm (gestational age < 37 weeks) compared to 33% (130/400) of inborn neonates (chi-square = 8.5, P-value <0.01).

**Table 2:** **Admission diagnoses and clinical characteristics of neonates admitted to Kiwoko Hospital NICU (December 2005 through September 2008)**

	All neonates		Inborn		Outborn	
	%	(No.)	%	(No.)	%	(No.)
**Sex**						
Male	55.2	(433)	55.4	(263)	55.5	(122)
Female	44.8	(352)	44.6	(212)	44.5	(98)
**Birth weight (kg)**						
<1	2.5	(18)	2.3	(11)	3.5	(6)
1-1.4	9.2	(67)	4.6	(22)	22.5	(39)
1.5-2.4	32.9	(240)	32.8	(157)	32.4	(56)
2.5-4	53.9	(393)	58.2	(279)	41.6	(72)
>4	1.5	(11)	2.1	(10)	0.0	(0)
**Gestational age (weeks)**						
<28	3.0	(20)	2.5	(10)	4.2	(8)
28-29	5.9	(39)	3.5	(14)	10.9	(21)
30-33	11.6	(77)	9.8	(39)	14.1	(27)
34-36	16.6	(110)	16.8)		15.6	(30)
37-42	60.0	(398)	64.8	(259)	51.6	(99)
>42	2.9	(19)	2.8	(11)	3.7	(7)
Admission diagnoses*						
Infection	29.8	(208)	24.3	(109)	47.9	(90)
Prematurity	29.5	(206)	26.8	(120)	32.5	(61)
Respiratory distress	28.3	(198)	37.3	(167)	8.5	(16)
Asphyxia	22.0	(154)	27.7	(124)	8.0	(15)
Jaundice	3.0	(21)	2.5	(11)	5.3	(10)
**Therapies received**						
Formal phototherapy	29.0	(148)	26.7	(84)	33.6	(48)
Oxygen therapy	60.7	(388)	61.6	(244)	54.4	(93)
Surgery	2.8	(15)	1.5	(5)	6.2	(9)
**Age at admission/hospital course (days)**	Median	(Range)	Median	(Range)	Median	(Range)
Age at admission (n = 809)	0	(0–28)	0	(0–28)	2	(0–28)
Duration of stay (n = 589)	6	(0–96)	5	(0–96)	7	(0–63)

*Percent from among the 699 neonates with at least one admission diagnosis provided; 448 inborn neonates and 188 outborn neonates. Note, neonates could have more than one admission diagnosis.

Each analysis is based on available data for that measure, as such denominators differ depending on the response rate.

**Figure 3:**
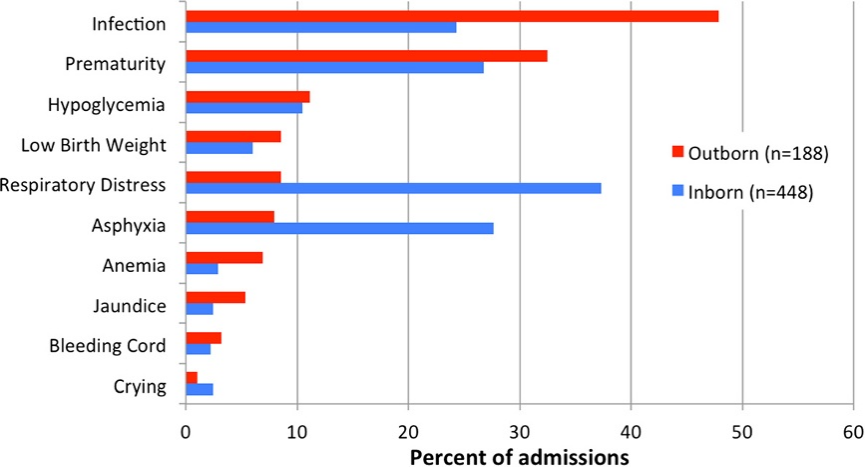
**Admission diagnoses by birth location.**

The most common diagnoses were infection, prematurity, respiratory distress, and asphyxia (Table 2), however differences in the relative frequency of these diagnoses between inborn and outborn neonates existed (Figure 3). Among outborn neonates the most common admission diagnosis was infection (48% (90/188)) whereas relatively few (8%, 16/188) were admitted for respiratory distress. In comparison inborn neonates were most commonly admitted for respiratory distress (37%, 167/448) and less commonly for infection (24%, 109/448). Sixty one percent (388/639) of patients received supplemental oxygen therapy, 29% (148/511) received phototherapy, and 3% (15/541) required surgery during their NICU course. Median age at admission for outborn infants was 2 days of age, and the median length of stay for all neonates was 6 days.

### Neonatal mortality

Survival to discharge among all neonates was 78% (578/745) (Table 3). Outborn neonates had almost twice the mortality (33%, 68/208) compared to inborn neonates (17%, 77/456) (chi-square = 20.9, P < 0.01). Median length of stay for neonates was 6 days for babies who survived to discharge and 5 days for those who did not. Mortality was inversely proportional to birth weight (P-value for trend = <0.01), with death occurring in 89% (16/18) of neonates with birth weight less than 1 kg (Table 4). In analyses disaggregated by birth location, mortality was inversely associated with birth weight among both inborn (P-value for trend <0.01) and outborn infants (P-value for trend <0.01). Mortality was also inversely associated with gestational age group overall and among inborn and outborn infants (P-value for trend <0.01 in each group), with greater mortality among younger gestational age groups.

**Table 3:** **Discharge status and outcome of neonates admitted to Kiwoko Hospital NICU (December 2005 through September 2008)**

	All neonates		Inborn		Outborn	
	%	(No.)	%	(No.)	%	(No.)
**Discharge status**						
Alive at discharge	77.6	(578)	83.1	(379)	67.3	(140)
Dead at discharge	22.4	(167)	16.9	(77)	32.7	(68)
	Median	(Range)	Median	(Range)	Median	(Range)
**Alive at discharge (days) (n = 447)**						
Duration of stay	6	(0–96)	5	(0–96)	7	(0–52)
Age at discharge	8	(0–96)	6	(0–96)	15	(1–52)
**Dead at discharge (days) (n = 140)**						
Duration of stay	5	(0–63)	5	(0–50)	6	(0–63)
Age at death	6	(0–64)	5	(0–50)	7	(0–64)

Each analysis is based on available data for that measure, as such denominators differ depending on the response rate.

**Table 4:** **Mortality by birth weight/ gestational age**

	All neonates		Inborn		Outborn	
	%	(No.)	%	(No.)	%	(No.)
**Birthweight**						
<1 kg	88.9	(16)	81.8	(9)	100.0	(6)
1.0-1.4 kg	68.9	(42)	57.1	(12)	74.3	(26)
1.5-2.4 kg	26.7	(58)	21.4	(31)	38.0	(19)
2.5-4 kg	9.9	(36)	8.9	(23)	12.1	(8)
>4 kg	0.0	(0)	0.0	(0)	0.0	(0)
**Gestational Age**						
<28 weeks	75.0	(15)	70.0	(7)	75.0	(6)
28-29 weeks	59.5	(22)	30.8	(4)	71.4	(15)
30-33 weeks	50.0	(33)	44.4	(16)	61.9	(13)
34-36 weeks	16.8	(17)	13.1	(8)	24.1	(7)
37-42 weeks	12.2	(46)	8.5	(21)	20.9	(19)
>42 weeks	5.3	(1)	9.1	(1)	0.0	(0)

Each analysis is based on available data for that measure, as such denominators differ depending on the response rate.

## Discussion

Our study reports specific demographics, diagnoses and gestational age/birthweight specific mortality for neonates admitted to a rural NICU. These data add to the limited literature on these important determinants of neonatal survival. Results such as these are the first step to targeting improvements to facility based care of neonates in LMIC settings.

We found that mortality rates were almost twice as high in outborn as compared to inborn neonates admitted to this Ugandan NICU. This increased mortality is likely due to risk factors disproportionately affecting outborn babies admitted to the NICU and is in line with previous studies in LMIC NICUs [21–23]. Outborn infants were more likely to be premature and/or low birth weight, delivered by a traditional birth attendant, and to have an infection than neonates born at the hospital. Delays in accessing a facility able to provide quality, appropriate, neonatal care may have reduced survival among outborn newborns in our study, consistent with the report by Lawn et al. on LMIC home births and intrapartum-related neonatal mortality [24]. Our findings support improving referral systems and facility-based care for sick outborn infants as a crucial part of the continuum of care necessary to decrease early neonatal mortality. This may be especially relevant in rural settings where access to quality neonatal care is most challenging.

Our results reiterate the detrimental effect which low birth weight and preterm birth have on neonatal survival in LMIC settings whether an infant was born in or out of the hospital. We found mortality was inversely correlated with gestational age and birth weight among our population. Improvements in access to prenatal care, and interventions to prevent preterm delivery and low birth weight are urgently needed; however, our findings suggest that improvements in facility based neonatal care may also offer a survival advantage. While mortality was very high for the smallest and most premature infants born (below 1 kg or 28 weeks gestation), this group accounted for only 2-3% of all admissions. Survival was markedly increased (73%) among infants between 1.5 and 2.5 kg, with this group making up a sizable proportion of all NICU admissions (33%). This improved survival in low birthweight (<2.5 kg) infants cared for in the NICU is encouraging given that globally, 16% of all newborns are low birthweight, and account for 60-80% of neonatal deaths [25, 3]. Additionally, since prematurity is disproportionately increasing as a cause of global childhood mortality, focusing on referral and facility-based newborn care interventions of the low birthweight infant may offer a feasible way to improve survival in high risk newborns [8].

Our study has several limitations. This study describes a hospital-based population of neonates whose mothers’ had sufficient resources to bring themselves or their baby to the hospital and does not therefore describe population-based mortality for the region. Those neonates admitted to the NICU may not be representative of all neonates born in the region, given that approximately one third of births happen in the home [18]. The differences in mortality between inborn and outborn babies who were admitted to the NICU may in part be attributable to multiple selection biases. A family caregiver’s access to transportation and their selection of which infant should be transported (i.e., parental triaging) may have influenced whether or not an outborn infant was brought to the NICU. The most severe outborn cases may not survive long enough to be transported. Only infants that survived transport and were admitted to the NICU, not infants that died en route, were included in our study. Finally, differences in delays to receiving appropriate therapy, and conditions during transport may have affected outcomes in outborn infants.

Another study limitation was the large amount of missing data in our sample. This limitation is not surprising given that nurses were providing patient care while collecting data, likely inhibiting their ability to focus on data entry. Readmissions to the NICU were not noted and while these were rare, some neonates may have been represented more than once. Additionally the unit of analysis was the infant and therefore mothers of twins are represented twice in our maternal characteristics results. This sample is also limited by an overall small number of infants within each subgroup, especially for gestational ages below 30 weeks and birth weights below 1.5 kg. Ascertainment of exact gestational age was a challenge in this study. For example, there were a surprisingly high proportion of births which occurred at exactly 40 weeks gestation in our dataset (not shown). This may represent a high proportion of women who reported their best estimate of the due date, as many women were not able to recall their last menstrual period, did not have prenatal care to estimate their due date, and/or the Ballard score was not always performed or was not always accurate. Finally, it should be noted that these data are now six to nine years old and standards of care and global awareness of newborn mortality have changed since that time.

## Conclusions

We report the distribution of key demographic and clinical characteristics within a rural Ugandan NICU and the variable burden of mortality among gestational age, birthweight and birth location specific groups. These results provide important baseline data for future research investigating the impact of targeted facility based newborn health interventions on mortality outcomes in a rural LMIC setting. This information is vitally important to assess interventions to improve global neonatal mortality as we reach the deadline for millennium development goal #4.

## Abbreviations

LMIC: Low and middle income countries
NICU: Neonatal Intensive Care Unit.
